# *In vivo* Effects of Disease-modifying Therapies on Immunological Subsets in Patients with Relapsing-Remitting Multiple Sclerosis

**DOI:** 10.2174/011570159X360729250319064959

**Published:** 2025-07-04

**Authors:** Chiara Finocchiaro, Clara Grazia Chisari, Salvatore Lo Fermo, Emanuele D’Amico, Nunziatina Laura Parrinello, Alessandra Romano, Giuseppe Alberto Palumbo, Sara Marino, Anna Maria Corsale, Francesco Di Raimondo, Mario Zappia, Francesco Patti

**Affiliations:** 1 Department “GF Ingrassia” Section of Neurosciences, University of Catania, Catania, Italy;; 2 UOS Multiple Sclerosis, Neurology Clinic, “G.Rodolico-San Marco” University Hopsital, Catania, Italy;; 3 Department of Clinical and Experimental Medicine, University of Foggia, Foggia, Italy;; 4 Hematology with BMT Unit, A.O.U. Policlinico “G.Rodolico-San Marco”, Catania, Italy;; 5 Dipartimento di specialità Medico-Chirurgiche, CHIRMED, Sezione di Ematologia Università degli Studi di Catania, Catania, Italy (Department of Medical-Surgical Specialties, CHIRMED, Hematology Section University of Catania, Catania, Italy);; 6 Department of Health Promotion, Mother and Child Care, Internal Medicine and Medical Specialties, University of Palermo, Palermo, Italy;; 7 Department of General Surgery and Medical-Surgical Specialties, University of Catania, Catania, Italy

**Keywords:** Multiple sclerosis, disease modifying therapies, cladribine, dimethyl fumarate, natalizumab, immunophenotype, myeloid cells, B cells, T cells

## Abstract

**Background:**

Disease-modifying therapies (DMTs) are aimed at controlling Multiple Sclerosis disease by modulating or suppressing the immune system. However, there is limited data on changes in immune cell subsets induced by these treatments.

**Objective:**

To assess differences in myeloid, T-, and B-cell subsets in the peripheral blood of relapsing-remitting MS (RR-MS) patients treated with different DMTs.

**Methods:**

This longitudinal study enrolled all RR-MS patients treated with cladribine (CLAD), dimethyl fumarate (DMF), and natalizumab (NTZ) between July 2022 and September 2022. All patients underwent blood sample collection with flow cytometry at baseline (T0; before starting treatment) and 24 ± 3 months after treatment initiation (T1).

**Results:**

Forty-three RR-MS patients (83.7% women; mean age 34.7 ± 11.1 years; median EDSS: 2.0, IQR: 1.0-2.8) were enrolled. Among them, 24 (55.8%) were treated with DMF, 10 (23.3%) with NTZ, and 9 (20.9%) with CLAD. At T1, patients assigned to CLAD showed a reduction in B-cell memory-switched (*p* = .029), B-cell memory-unswitched (*p* = .08), and B-cell naïve resting (*p* = .029). Additionally, the T and NK cell compartments showed a reduction in the percentage of CD3/CD4/CD127/CD45RA/CD161+ (*p* = .057). In the NTZ group, a significant decrease in the percentage of CD3/CD4/CD127/CD45RA/CD161+ (*p* = .029) was observed. A reduced percentage of mature naïve B cells (*p* = .057) and B memory-unswitched (*p* = .059) was observed in the DMF group. No significant differences were found in the myeloid subsets.

**Conclusion:**

DMTs induced significant modifications in B- and T-cell compartments. Characterizing these immunologic changes could deepen our understanding of the mechanisms of action of different therapies.

## 1 INTRODUCTION

Various dependable biomarkers, such as MRI assessments, immunoglobulin G (IgG), oligoclonal bands (OCB), and neurofilament light chain (NfL) levels in cerebrospinal fluid (CSF), have been recognized for tracking treatment responses in Multiple Sclerosis (MS) patients [1]. However, these biomarkers offer limited insights into the immunological changes underlying MS pathogenesis [2]. The movement of activated immune cells from the periphery into the central nervous system (CNS) drives aberrant immune responses in MS. Thus, peripheral blood could be a more readily available biological sample for exploring MS immunopathogenesis and could be a target for specific therapeutic interventions [3].

Although multiparametric flow cytometry technologies have advanced recently, information on alterations in immune cell subsets within the peripheral blood of MS patients is still limited. In autoimmune diseases, cancer, infections, trauma, and graft-*versus*-host disease, myeloid-derived suppressor cells (MDSCs) are known to expand, leading to immune dysfunction as a result of cytokine overload and abnormal myelopoiesis [4]. MDSCs are immature myeloid cells that diverge from typical differentiation pathways [5]. Based on their phenotypic and morphological features, MDSCs can be categorized into granulocytic or polymorphonuclear (G/PMN-MDSC), which resemble neutrophils, and monocytic (Mo-MDSC), which are akin to monocytes and macrophages. The main feature of these cell subtypes is their ability to suppress the immune system, especially by targeting T cells [6]. During early MS stages, research indicates that monocytes and macrophages are key myeloid cell types involved in mediating both pro-inflammatory and anti-inflammatory responses [7-10]. Moreover, MS patients exhibited a notable increase in the CD16+ monocyte population in peripheral blood compared to healthy controls (HCs), primarily driven by intermediate and nonclassical monocytes with pro-inflammatory activity, alongside a corresponding decrease in the classical monocyte subset [11]. In a recent case-control study, myeloid, B-, and T-cell populations were assessed using flow cytometry in naïve relapse-remitting MS (RR-MS) patients and HCs, revealing distinct immunophenotypic characteristics in naïve MS patients [12].

Several studies have investigated the effects of disease-modifying therapies (DMTs) on peripheral blood immune cell populations in MS patients, highlighting significant changes [13-15]. Most of these therapies have been shown to alter the composition and function of various immune cells. For instance, fingolimod significantly reduces lymphocyte levels, while other DMTs affect both B and T cell compartments, influencing overall immune responses, including those to vaccines like the SARS-CoV-2 vaccine [16]. On the other hand, fingolimod reduces B-cell abundance in the blood, while dimethyl fumarate (DMF) and glatiramer acetate have minimal effects on B-cell numbers but influence cytokine production differently [17]. Another study found that natalizumab (NTZ) promotes the activation and pro-inflammatory differentiation of peripheral B cells in MS patients, increasing the number of B cells compared to untreated controls [18]. The research examined the impact of various DMTs on peripheral blood mononuclear cells (PBMCs) from MS patients, finding alterations in the expression profiles of immune checkpoints and co-stimulatory molecules, which are crucial for the immune response regulation in MS [19]. Additionally, DMTs like DMF and natalizumab can modulate the functions of dendritic cells, which are crucial for initiating and regulating immune responses. The effects on these and other innate immune cells suggest that DMTs exert broad immunomodulatory actions beyond their immediate targets [17].

Despite these advancements, gaps remain in the complete understanding of the differential effects of specific DMTs on immune cell subsets. Our study aims to address these gaps by evaluating immunophenotypic changes in RR-MS patients treated with DMF, NTZ, and cladribine (CLAD).

## 2 MATERIALS AND METHODS

### 2.1 Study Design and Settings

This longitudinal *in vivo* study was conducted at the MS Center of the University of Catania, Italy, from July to September 2022, aiming to assess the impact of various DMTs on T- and B-lymphocytes, as well as myeloid cells, in the peripheral blood of patients with RR-MS. We selected patients receiving treatment with DMF, NTZ, and CLAD, each of which operates through distinct mechanisms.

Specifically, DMF is administered orally and activates the nuclear factor erythroid 2-related factor 2 (Nrf2) pathway, which modulates enzymes to mitigate oxidative stress [20]. NTZ, a monoclonal antibody, hinders autoreactive leukocytes from leaving blood vessels and infiltrating target organs to trigger inflammation [14]. CLAD, a purine analogue, disrupts DNA synthesis, leading to a decrease in circulating B and T lymphocytes [21].

Neurologists prescribed DMF, CLAD, and NTZ according to the patient's disease profiles (including relapse frequency, EDSS, and MRI findings) and the eligibility criteria set by the Agenzia Italiana del Farmaco (AIFA).

The study population consisted of patients diagnosed with RR-MS based on the 2017 McDonald criteria. The study enrolled patients who met the following inclusion criteria: (1) aged 18 to 65 years; (2) diagnosed with RR-MS; (3) either treatment-naïve or switched from another first-line therapy; and (4) possessing adequate organ function. Exclusion criteria included: (1) significant cardiovascular, pulmonary, gastrointestinal, hepatic, or renal conditions; (2) a history of cancer; (3) severe psychiatric disorders; (4) recent infections necessitating systemic antibiotic treatment within 4 weeks before study entry; and (5) pregnancy or breastfeeding.

All patients who experienced a clinical and/or radiological relapse between T0 and T1 were excluded from the study, as well as those who received steroid therapy or other treatments for other reasons that could potentially influence the cytometric evaluations.

All patients underwent clinical and radiological assessments, followed by blood sample collection at baseline (T0; before starting treatment) and 24 ± 3 months after treatment initiation (T1).

The flow cytometry analysis was assessed at the Hematology Centre of the University of Catania, Italy.

The study was approved by the Policlinico-Vittorio Emanuele (Catania, Italy; cod PO/898/2021) Ethics Committee.

### 2.2 Clinical Assessment and Neuroimaging

We collected the following demographic, clinical, and radiological data: (a) demographic (age and gender), (b) clinical (disease duration, changes in disability assessed by the Expanded Disability Status Scale (EDSS) and number of relapses in the months between diagnosis and follow-up), (c) MRI (number of brain and spinal cord lesions in T2 weighted and T1 gadolinium-weighted sequences at baseline and follow-up), and (d) DMTs (type of DMT and treatment duration).

Clinical evaluations involved recording the EDSS score at the time of diagnosis and during a follow-up assessment conducted within 6 months after the second blood sample was taken.

MRI was performed at the time of diagnosis (baseline MRI) and within 6 months of the second blood sample collection (follow-up MRI). All MRI scans were obtained using the 1.5-Tesla MRI.

### 2.3 Multidimensional Flow Cytometry (MFC)

All patients underwent peripheral blood collection; blood samples were collected in ethylenediaminetetraacetic acid (EDTA) tubes between 8:30 am and 10:30 am and processed within 2 hours. Three mL of peripheral blood were collected in EDTA tubes. Samples were stained following the EuroFlow lyse, wash and stain standard sample preparation protocol, adjusted to 106 nucleated cells, using the following tubes for standardized staining (Table **S1**): Tube 1, for the evaluation of the myeloid compartment: CD15, CD33, CD38, CD64, HLA-DR, PD-L1, CD14, CD16, CD45; Tube 2 for T- and Natural Killer lymphocytes detection: CD3, CD4, CD161, CD127, CD16, PD-1, CD45RA, CD45; Tube 3, Dura Clone IM B Cells kit (Beckman Coulter), a reagent panel of 8 monoclonal antibodies for B--cell detection: IgD, CD21, CD19, CD27, CD24, CD38, IgM, CD45.

In each tube, 50 µL of peripheral blood was transferred and incubated for 15 minutes at room temperature in darkness, following the manufacturer’s instructions. Unstained controls were used to set the flow cytometer photomultiplier tube voltages, and single-color positive controls were used to adjust instrument compensation settings. Precision Count Beads™ were used to obtain absolute counts of cells acquired by flow cytometer. Data from stained samples were acquired using a Beckman Coulter Navios EX-10 flow cytometer and analyzed using Kaluza™ Software 2.1 (https://www.beckman.it/flow-cytometry/software/kaluza-c).

### 2.4 Computational Flow Cytometry

Flow cytometry datasets from MS patients, obtained both before and after treatment, were processed using the semi-automated algorithm “FlowCT” v.0.0.951. This recently developed workflow is intended to unravel immunophenotypic data and provide an objective analysis of large datasets by processing multiple files through automated cell clustering. [22-24]. FlowCT employs a four-step bioinformatics approach: (1) data evaluation (including normalization and cleaning), (2) clustering and dimensionality reduction utilizing FlowSOM (version 1.14.1) and UMAP, (3) manual cluster annotation based on characteristic markers, and (4) statistical analysis. Briefly, FCS files were merged, subjected to quality control, normalized to remove batch effects, and clustered using FlowSOM. Following computational clustering, each cluster was identified using Infinicyt software v.2.0 (Cytognos SL, Salamanca, Spain, https://www.cytognos.com). The immune cell composition of peripheral blood from MS patients, encompassing T, B, and NK cells, was assessed using FlowCT, a semi-automated workflow optimized for the efficient analysis of large datasets. In summary, the samples underwent pre-processing, multiple dimensionality reduction methods, clustering *via* FlowSOM, population identity assignment, and statistical comparison to evaluate the abundance of each identified cell population.

Details of the monoclonal antibodies used in the study were illustrated in Table **S1**.

### 2.5 Monoclonal Antibodies

Myeloid cell subsets were characterized using a panel of nine monoclonal antibodies: CD15-FITC (80H5), CD14-PE (RMO52), CD64-ECD (22), CD16-PC5 (3G8), PD-L1-PC7 (PD-L1.3.1), CD33-APC (D3HL60.251), CD38-A750 (LS198-4-3), HLADR-PB (Immu-357) and CD45-KO (J33).

A panel of nine monoclonal antibodies was also used in order to identify T-cell subsets: PD1-FITC (PD1.3.1.3), CD127-PE (R34.34), CD3-ECD (UCHT1), CD8 FITC (B9.11), CD25-PC5 (B1.49.9), CD4-PC7 (SFCI12T4D11), CD161-ALEXA750 (191B8), CD45RA-PB (2H4LDH11-LDB9) and CD45-KO (J33).

Lastly, a panel of eight monoclonal antibodies and the Dura Clone IM B Cell kit (Beckman Coulter) were used in order to phenotypically evaluate B-cell populations: IgD (FITC), CD21 (PE), CD19 (ECD), CD27 (PC7), CD24 (APC), CD38 (APC-750), IgM (Pacific Blue) and CD45 (Krome Orange).

### 2.6 Immunological Cell Subset Definitions

#### 2.6.1 Lymphocytes

We detected CD3-CD16+ (natural killer, NK), CD3+CD4+ (T-helper), and CD3+CD8+ (T-cytotoxic) cells. Among the T-helper subset, we identified different subpopulations: CD4+CD25+CD127/low (T-regulatory), CD4+CD161+ and CD4+CD45RA+ (T-naïve) cells.

After CD19-positive cells gating, the differential expression of CD38, CD27, IgM and IgD permitted B-subpopulations’ discrimination: (CD19+) IgD+CD27- (B-naïve), (CD19+ IgD-IgM-) CD38-/CD27+ (switched B-memory) and (CD19+ IgD+IgM+) CD38-/CD27+ (unswitched B-memory) cells.

#### 2.6.2 Myeloid Cells

We distinguished cells expressing CD15+/CD33+/CD14-/ HLADR−/low, defined as G-MDSCs, and cells considered myeloid counterparts such as Mo-MDSCs CD14+/HLADR−/ low and inflammatory monocytes, CD14+CD16+.

### 2.7 Statistical Analysis

Statistical analyses were performed using STATA 16.1.Continuous variables were expressed as mean ± standard deviation (SD) or median with interquartile range (IQR). Categorical variables were expressed as frequencies and percentages. The Shapiro-Wilk test was used to assess the normality of the numerical data distribution. A paired t-test or Wilcoxon signed-rank test was used for within-group comparisons, and a one-way ANOVA or Kruskal-Wallis test was applied for between-group comparisons. If the distribution was non-parametric, the Kolmogorov-Smirnov test was applied for continuous variables, and the Mann-Whitney U-test for nominal variables. In order to compare categorical variables, Chi-square and Fisher exact tests were applied. Analysis of variance (ANOVA) was performed to assess the main and interaction effects among the three DMT groups. Bonferroni correction was applied for multiple post-hoc comparisons.

Correlation analyses were conducted between significantly different cell subsets and clinical/radiological measures, using Pearson and partial Pearson coefficients, adjusted for age and sex for continuous variables. Specifically, we examined the association between changes in immunophenotypic cell subsets from baseline to follow-up and corresponding clinical and radiological characteristics.

## 3 RESULTS

Out of 52 RR-MS patients screened, 43 (83.7% women; mean age at diagnosis 34.7 ± 11.1 years; median EDSS 2.0, IQR 1.0 - 2.8) were finally enrolled. Nine patients were excluded because they suspended the DMT because of efficacy or safety reasons. Of them, 24 (55.8%) were assigned to DMF treatment, 10 (23.3%) to NTZ and 9 (20.9%) to CLAD. Patient demographics and clinical and radiological features at baseline (T0) are illustrated in Table **1**. Particularly, patients assigned to NTZ and CLAD treatments showed a high number of brain and spinal lesions on T2 weighted sequences and of T1 gadolinium-enhanced lesions compared to those assigned to DMF.

Similarly, at follow-up (T1), a higher number of brain MRI lesions on T2 weighted sequences in the NTZ and CLAD patients, compared to DMF patients, was found (Table **2**).

Flow cytometry data considering different cell subset analyses were illustrated in Fig. (**1**).

**Fig. (2):**
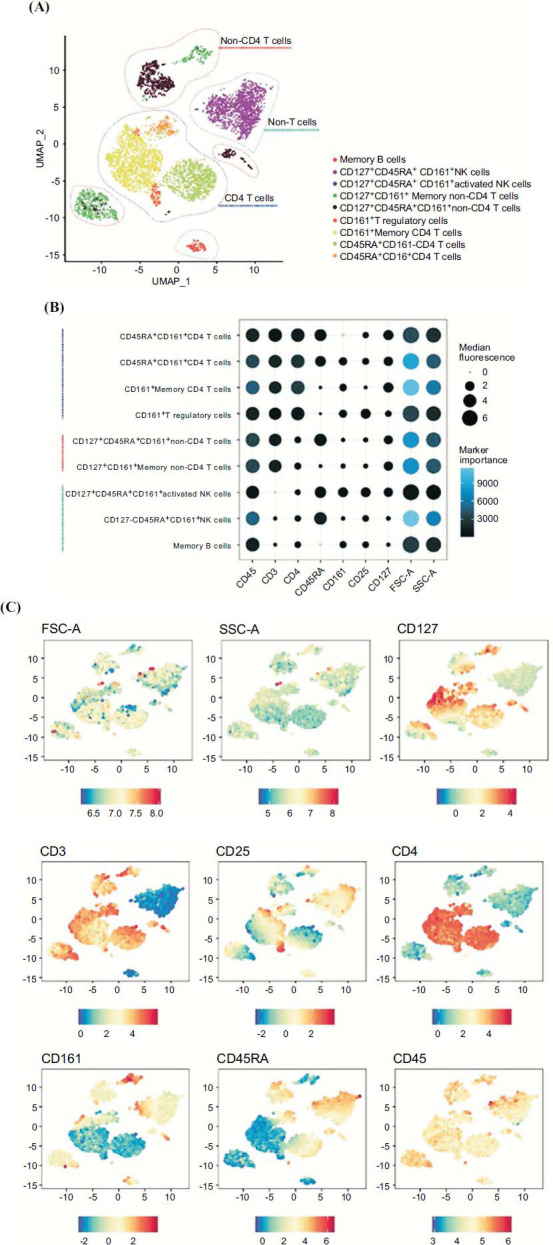
The immune landscape of peripheral blood cells from MS subjects undergoing specific treatment: a focus on T and NK cell compartments. (**A**) Uniform manifold approximation and projection (UMAP) of identified by self-organizing map (SOM) of T and NK cell compartments from all MS subjects included in the study. (**B**) Dot plot showed the relative expression levels of each marker (x-axis) across different clusters (y-axis), providing insight into the variations within the identified cell populations. (**C**) Uniform manifold approximation and projection (UMAP) of identified by self-organizing map (SOM) of T and NK cell compartments.

**Fig. (3):**
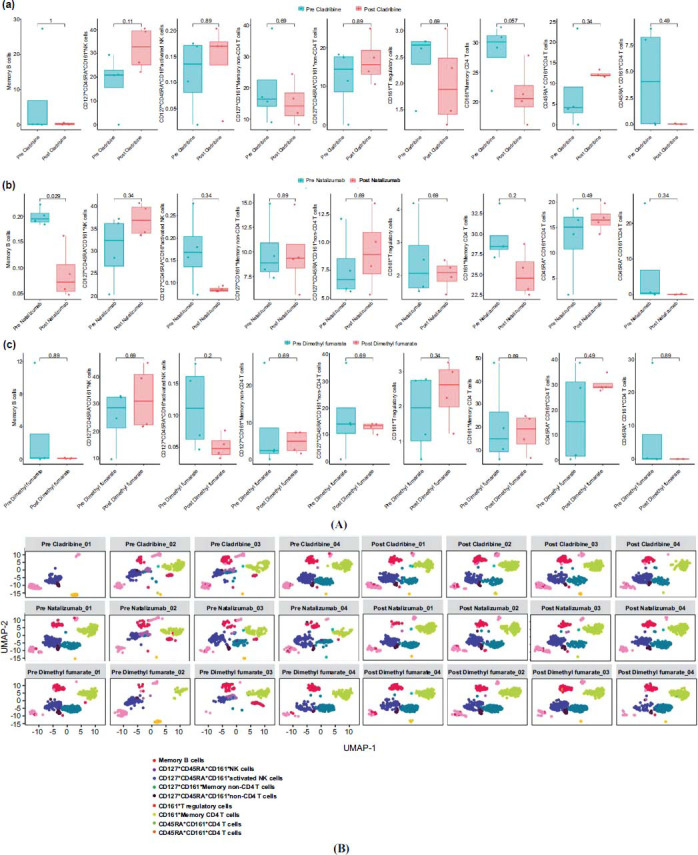
(**A**) Alteration in T and NK cell compartments in MS subjects undergoing specific treatment. Boxplots illustrated changes in the percentage of T and NK cells at baseline (blue bars) or after treatment (pink bars) with Natalizumab (**a**), Cladribine (**b**), Dimethyl fumarate (**c**). (**B**) Changes in T-cell compartment for representative patients. Uniform manifold approximation and projection (UMAP) of identified by self-organizing map (SOM) of T and NK cell compartments from all MS subjects at baseline and after treatment.

**Fig. (4):**
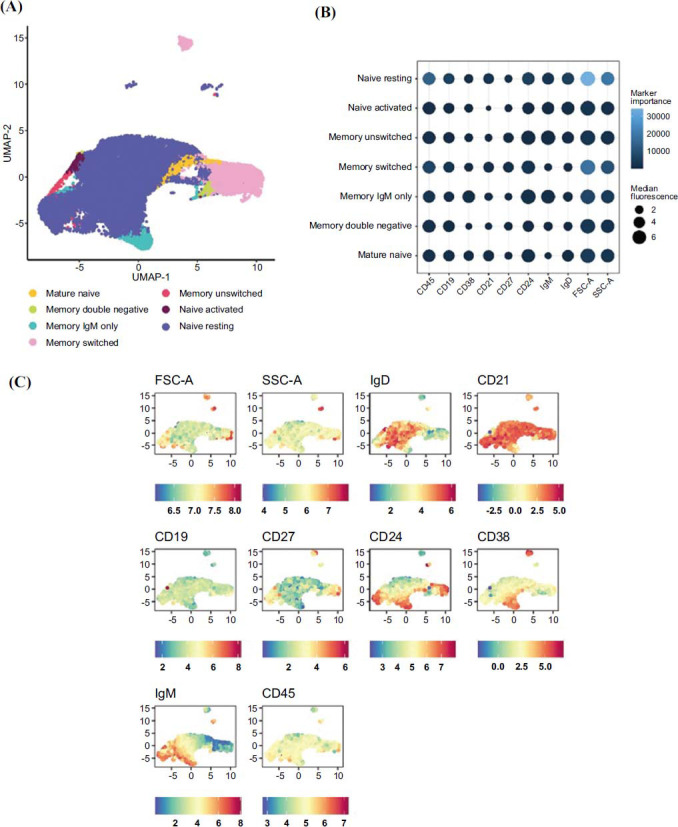
The immune landscape of peripheral blood cells from MS subjects undergoing specific treatment: a focus on B cell compartments. (**A**) Uniform manifold approximation and projection (UMAP) of identified by self-organizing map (SOM) of B-cell compartment from all MS subjects included in the study. (**B**) Dot plot showed the relative expression levels of each marker (x-axis) across different clusters (y-axis), providing insight into the variations within the identified cell populations. (**C**) Uniform manifold approximation and projection (UMAP) of identified by self-organizing map (SOM) of B-cell compartment.

**Fig. (5):**
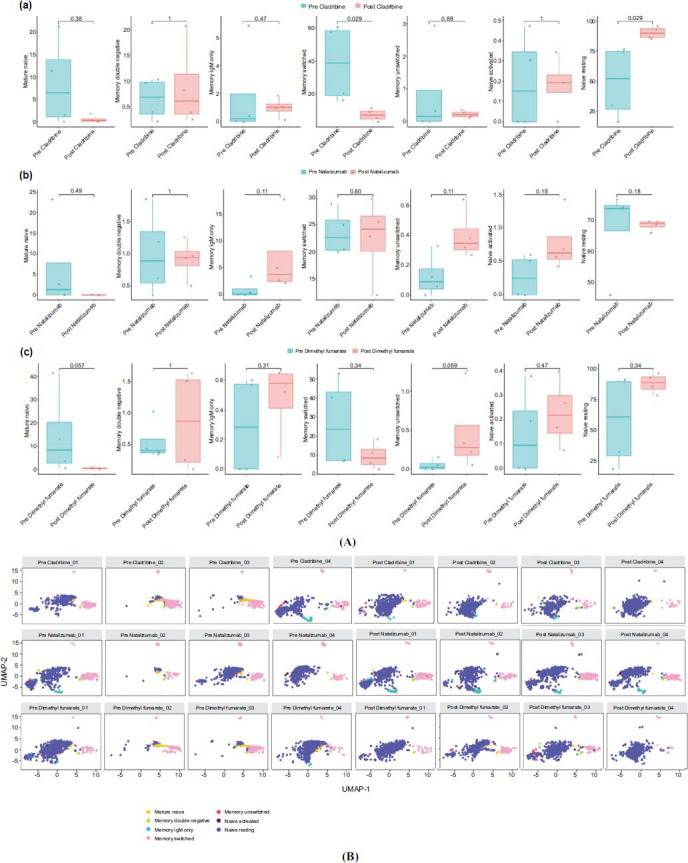
(**A**) Alteration in B cell compartments in MS subjects undergoing specific treatment. Boxplots illustrated changes in the percentage of B cells at baseline (blue bars) or after treatment (pink bars) with Natalizumab (**a**), Cladribine (**b**), Dimethyl fumarate (**c**). (**B**) Changes in B-cell compartment for representative patients. Uniform manifold approximation and projection (UMAP) of identified by self-organizing map (SOM) of B cell compartment from all MS subjects at baseline and after treatment.

At T1, in patients assigned to CLAD and NTZ treatments, T and NK cell compartments showed a reduction in the percentage of CD3 CD4 CD127 CD45RA CD161+ (*p* =.057 and *p* =.029, respectively) (Figs. **2**, **3A** and **3B**). In addition, a reduction in B cells memory switched (*p*=.029), B cells memory unswitched (p =.08), and of the B cells naïve resting (*p*=.029) was found in the CLAD group (Figs. **4**, **5A** and **5B**).

Patients treated with DMF showed a reduced percentage of mature naïve B cells (*p*=.057) and of B memory unswitched (*p*=.059), but these were not statistically significant (Figs. **4**, **5A**, and **5B**).

At T1, no significant differences were found in the myeloid subsets compared to baseline values.

No significant correlations were found between differences in terms of immunophenotypic cell subsets from baseline to follow-up and clinical/radiological characteristics (Table **S2**).

## 4 DISCUSSION

This study demonstrates that DMTs induce significant changes in B- and T-cell compartments in RR-MS patients. The reduction in B-cell subsets observed in CLAD-treated patients, and the decrease in CD3 CD4 CD127 CD45RA CD161+ cells in both the CLAD and NTZ groups suggest that these therapies exert strong immunomodulatory effects.

In our cohort, CLAD induced a marked reduction in B cells (memory-switched, memory-unswitched, and naïve-resting). This finding is consistent with the MAGNIFY-MS substudy, which reported a rapid decrease in CD19+, CD20+, memory, activated, and naïve B cells, reaching a nadir two months post-treatment [25]. Interestingly, in this study, total CD19+, CD20+, and naïve B cell counts were reconstituted, while memory B cells remained reduced by 93-87% [25]. Moreover, patients treated with CLAD in our cohort showed a decrease in the percentage of the T CD3+ lymphocytes (CD3 CD4 CD127 CD45RA CD161+). Activated CD3+ T cells penetrate the BBB, secrete pro-inflammatory cytokines, and trigger an autoimmune response against myelin components. Consequently, the stimulation of microglia, astrocytes, and B cells results in demyelination and neurodegeneration [26]. Hence, the finding of a reduction in the T CD3+ count could reflect the suppressive effect of CLAD. These results were in line with two other recent studies [27, 28]. In particular, a progressive decrease in the CD3+CD4+ and CD3+CD8+ T cell count, with the lowest levels at month 15 after therapy with oral CLAD, was found in 18 RR-MS patients [27]. Similarly, an *ex vivo* to *in vitro* study showed lower levels of the CD3+ cell in CLAD-treated MS patients compared to the untreated [28].

In our study, DMF caused an effect on several B cell subtypes, even if not statistically significant. Indeed, in line with the recent literature, patients treated with DMF showed an increase in the B naïve cells, a decrease in the switched B memory cells [29-33], and an increase in the percentage of the CD4+CD45RA+ (T naïve) [34]. In contrast, the CD3-CD16+ (NK) and the T cell compartments were not affected by treatment with DMF. This last result may be explained by their germinal center independence with consequent poly-reactivity maintenance [35]. Moreover, our results showed reduced levels of CD3+CD4+ (T-helper), CD3+CD8+ (T-cytotoxic), CD4+CD161+, and CD4+CD25+CD127low/− (T-reg), as observed in other studies [36-39]. It is known that T-reg lymphocytes induce immune tolerance by preventing activation of potential auto-reactive T cells in healthy subjects [40] but are less competent in suppressing CD4 + T cell proliferation in MS [41-43]. Thus, a decrease in their percentage after DMF treatment, in association with the other immunophenotypic changes, although not statistically significant, may suggest a reduction in the overall T and B cells' potential to migrate into the CNS and to induce immune activation and autoreactivity processes, that are typically associated with MS [44, 45].

According to our results, NTZ treatment induced a reduction in the percentage of T CD3+, while no modifications were observed in the percentage of the total B CD19+ lymphocytes, as demonstrated in other studies [29, 46-48]. The finding of the high percentage of B cells in peripheral blood reflected the NTZ mechanism of action of blocking the entrance of lymphocytes into the CNS with consecutive retention of these cells in the peripheral blood [49].

Notably, most of the significant results on the T cell compartment refer to the CD3 CD4 CD127 CD45RA CD161+ population. This subset of cells refers to a specific population of T-helper cells characterized by markers associated with both regulatory and pro-inflammatory immune responses in MS [3, 50].

Although aberrant myeloid function is often observed in MS, the majority of MS therapies focus on lymphocytes, and the effects of current DMTs on myeloid cells are currently overlooked. In our previous study, monocytic myeloid-derived suppressor cells (Mo-MDSCs CD14+/HLADR−/low) and inflammatory monocytes (CD14+CD16+) displayed higher frequencies in RRMS patients when compared with HCs. Nevertheless, in the present study, no significant modifications were found after treatment [12]. Recent studies conducted on both naïve and DMTs treated MS patients, compared to HCs, have shown conflicting and non-unique results [10, 51]. In particular, a study demonstrated a significant expansion of the CD16+ nonclassical monocyte population, accompanied by a proportionate reduction in the classical monocyte population, in MS (treated and untreated) patients compared to HCs [10]. Conversely, another study illustrated granulocytes and both classical and nonclassical monocytes expanding in inactive RR-MS patients as compared with HCs and other MS forms [51]. In addition, several studies agree that alterations of intermediate or nonclassical monocyte levels are common in inflammatory diseases, such as MS, and the characterization of these monocyte subsets could help to clarify MS pathogenetic mechanisms and facilitate specific therapeutic development [52, 53]. Thus, selective monocyte subset depletion could represent a possible novel therapeutic approach [54]. These discrepancies may stem from the heterogeneity of the analyzed groups. Indeed, factors such as differences in disease duration, prior treatments, and baseline immune profiles can significantly influence outcomes. Addressing this heterogeneity is crucial for interpreting immunophenotypic changes and developing tailored therapeutic strategies.

Finally, no correlations were found between immunophenotype changes and clinical and radiological parameters. Although the immune deviation was not associated with effectiveness parameters, immunomonitoring by flow cytometry, together with clinical and imaging data, could deepen our knowledge about the mechanism of action of different DMTs.

Our study has some limitations. First, the small sample size of the cohort may have limited the generalizability of the results. Second, immunophenotyping studies have intrinsic methodological limitations, including normal inter-individual variations in the immune system, as well as heritable and non-heritable influences from microbial and environmental factors. Moreover, while we did not measure cytokine levels and their correlation with cell alteration, the observed modifications in T and B cell populations suggest that DMTs have significant immunomodulatory effects in RRMS patients, which may support the existence of an autoimmune inflammatory response to these DMTs.

On the other hand, our study has several strengths: first of all, we did not only focus on lymphocyte subsets but also provide an in-depth evaluation of myeloid cell populations, which have been less extensively studied in the context of MS and its treatment with DMTs. Specifically, we assessed changes in MDSCs and monocyte subsets, which are crucial for understanding the comprehensive immunological landscape of MS. Secondly, we recognize that the sample size is a limitation, particularly for the NTZ and CLAD groups. Despite this, our findings contribute valuable insights into the differential impacts of these therapies on immune cell populations. Increasing the sample size in future studies will be a priority to validate and expand upon these findings, enhancing the robustness of the results.

## CONCLUSION

In conclusion, our *in vivo* study adds to the current literature by providing a detailed analysis of both lymphoid and myeloid cell changes in MS patients undergoing different DMTs. Although much has been published on the impact of DMTs on the peripheral immune system in MS, our study provides novel insights into the specific immunophenotypic changes induced by CLAD, DMF, and NTZ. The detailed characterization of these changes enhances our understanding of the differential mechanisms of action of these therapies. We believe that this comprehensive immunophenotypic profiling is essential for a more complete understanding of the immunological mechanisms at play in MS and the effects of its treatments. Further research with larger cohorts will undoubtedly strengthen the conclusions and impact of this work.

## Figures and Tables

**Fig. (1):**
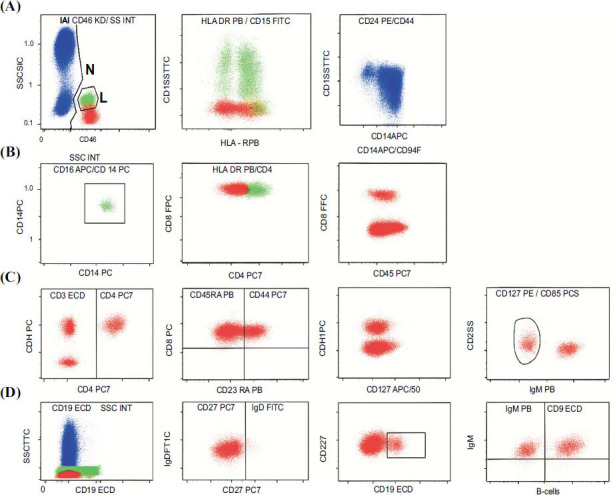
Flow cytometry data showing different cell subset analyses. Initial Gating: CD45 KO *vs.* SS INT to identify lymphocytes (L), monocytes (M), and neutrophils (N). (**A**) Myeloid Cells: HLADR PB *vs.* CD15 FITC to identify HLADR+ cells. CD33 APC *vs.* CD16 PE for HLADR+ cells. (**B**) Monocytic Cells: CD14 PE *vs.* CD64 ECD to identify monocytes. HLADR PB *vs.* CD16 PE for CD14+CD64+ cells. (**C**) T-Cells: CD3 ECD *vs.* CD4 PC7 to identify CD4+ T-cells. CD45RA PB *vs.* CD4 PC7 for CD4+ T-cells. CD161 APC *vs.* CD4 PC7 for CD4+ T-cells. CD127 PE *vs.* CD25 PCS for CD4+ T-cells. (**D**) B-Cells: CD19 ECD *vs.* SS INT to identify B-cells.CD27 PC7 *vs.* IgD FITC for CD19+ B-cells. IgM PB *vs.* IgD FITC for CD19+ B-cells.

**Table 1:** Patients demographics, clinical and radiological features at baseline.

**N (%)**	**Total** **43**	**DMF** **24 (55.8)**	**NTZ** **10 (23.3)**	**CLAD** **9 (20.9)**	***p*-value Anova** **(Post-Hoc Analysis)**
Female n (%)	36 (83.7)	18 (75)	9 (90)	7 (77.8)	ns
Age at diagnosis (year), (mean ± SD)	34.7 ± 11.1	33.5 ± 11.5	38.1 ± 11	34.2 ± 10.7	ns
Baseline EDSS (median, IQR)	2.0 (1.0 - 2.8)	1.3 (0.8-2.1)	2.5 (1.6-3.9)	2.0 (1.5-3.5)	NTZ *vs*. DMF: .08
No. of brain baseline MRI lesions on T2 weighted sequences (mean ± SD)	24.3 ± 20.1	15.8 ± 16.8	33.1 ± 18.9	37.4 ± 20.1	NTZ *vs*. DMF: .04 CLAD *vs*. DMF: .01
No. of spinal baseline MRI lesions on T2 weighted sequences (mean ± SD)	2.4 ± 2.3	1.6 ± 2.1	3.5 ± 2.4	3.2 ± 2	NTZ *vs*. DMF: .08
No. of brain baseline MRI lesions on T1 gadolinium weighted sequences (mean ± SD)	1.6 ± 5.5	0.4 ± 1	4.4 ± 11	1.8 ± 2.2	NTZ *vs*. DMF: .01
No. of spinal baseline MRI lesions on T1 gadolinium weighted sequences (mean ± SD)	0.3 ± 0.6	0.3 ± 0.5	0.6 ± 0.8	0.3 ± 0.5	ns

**Abbreviations**: CLAD: cladribine; DMF: dimethyl fumarate; EDSS: Expanded Disability Status Scale; IQR: interquartile range; MRI: Magnetic Resonance Imaging; NTZ: natalizumab; SD: standard deviation.

**Table 2:** Patients demographics, clinical and radiological features at follow-up.

**N (%)**	**Total** **43**	**DMF** **24 (55.8)**	**NTZ** **10 (23.3)**	**CLAD** **9 (20.9)**	***p* – value Anova** **(Post-Hoc Analysis)**
Age at follow-up (year), (mean ± SD)	36.4 ± 11.1	35.3 ± 11.6	39.8 ± 10.8	35.9 ± 10.7	ns
Patients with relapses in the months between naïve and follow-up PB sample n (%)	9 (21)	5 (21)	0	4 (44.4)	ns
Follow-up EDSS (median, IQR)	1.0 (0-2.0)	1.0 (0-1.6)	1.5 (0.3-2.3)	1.5 (1.0-2.0)	ns
No. of brain follow-up MRI lesions on T2 weighted sequences (mean ± SD)	27 ± 19.2	17 ± 11.9	36.4 ± 19.6	43.1 ± 19.8	NTZ *vs*. DMF: .006 CLAD *vs*. DMF: < .001
No. of spinal follow-up MRI lesions on T2 weighted sequences (mean ± SD)	3.1 ± 2.4	1.9 ± 2.2	4.9 ± 1.6	4.3 ± 2	NTZ *vs*. DMF: .001 CLAD *vs*. DMF: .01
No. of brain follow-up MRI lesions on T1 gadolinium weighted sequences (mean ± SD)	0 ± 0.3	0	0	0.2 ± 0.7	ns
No. of spinal follow-up MRI lesions on T1 gadolinium weighted sequences (mean ± SD)	0 ± 0.2	0 ± 0.2	0	0	ns

**Abbreviations**: CLAD: cladribine; DMF: dimethyl fumarate; EDSS: Expanded Disability Status Scale; IQR: interquartile range; MRI: Magnetic Resonance Imaging; NTZ: natalizumab; SD: standard deviation.

## Data Availability

The data and supportive information are available within the article.

## LIST OF ABBREVIATIONS

CNS: Central Nervous System
CSF: Cerebrospinal Fluid
DMF: Dimethyl Fumarate
DMTs: Disease-modifying Therapies
HCs: Healthy Controls
IgG: Immunoglobulin G
MFC: Multidimensional Flow Cytometry
MS: Multiple Sclerosis
MDSCs: Myeloid-derived Suppressor Cells
OCB: Oligoclonal Bands
PBMCs: Peripheral Blood Mononuclear Cells

## STANDARDS OF REPORTING

STROBE guidelines were followed.
