# Supplementary material for: In vivo Effects of Disease-modifying Therapies on Immunological Subsets in Patients with Relapsing-Remitting Multiple Sclerosis
Source: Curr Neuropharmacol. 2025 Jul 4;23(13):1783–96. doi: 10.2174/011570159X360729250319064959 (PMC12645145; doi:10.2174/011570159X360729250319064959)
Supplement: Supplementary file 1 [file CN-23-13-1783_SD1.pdf]

Supplementary Material

In vivo Effects of Disease-modifying Therapies on Immunological Subsets in Patients with Relapsing-Remitting Multiple Sclerosis

Chiara Finocchiaro[1,2,#], Clara Grazia Chisari[1,2,#], Salvatore Lo Fermo[2], Emanuele D'Amico[3], Nunziatina Laura Parrinello[4], Alessandra Romano[4,5], Giuseppe Alberto Palumbo[4,6], Sara Marino[4], Anna Maria Corsale[7], Francesco Di Raimondo[4,8], Mario Zappia[1] and Francesco Patti[1,2,*]

[1]Department "GF Ingrassia" Section of Neurosciences, Section of Neurosciences, University of Catania, Catania, Italy; [2]UOS Multiple Sclerosis; Neurology Clinic; "G.Rodolico-San Marco" University Hopsital, Catania, Italy; [3]Department of Clinical and Experimental Medicine, University of Foggia, Foggia, Italy; [4]Hematology with BMT Unit, A.O.U. Policlinico "G.Rodolico-San Marco", Catania, Italy; [5]Dipartimento di specialità Medico-Chirurgiche, CHIRMED, sezione di Ematologia Università degli Studi di Catania, Catania, Italy; (Department of Medical-Surgical Specialties, CHIRMED, Hematology Section University of Catania, Catania, Italy); [6]Department "GF Ingrassia" Section of Neurosciences, University of Catania, Catania, Italy; [7]Department of Health Promotion, Mother and Child Care, Internal Medicine and Medical Specialties, University of Palermo, Palermo, Italy; [8]Department of General Surgery and Medical-Surgical Specialties, University of Catania, Catania, Italy

1875-6190/25 $65.00+.00 **© 2025 Bentham Science Publishers**

Table S1. Comparisons between baseline and follow-up total immunological subsets.

N (%)	*Total Baseline 43*	*Total Follow-up 43*	*p value*	*DMF Baseline 24 (55.8)*	*DMF follow-up 24 (55.8)*	*p value*	*NTZ baseline 10 (23.3)*	*NTZ follow-up 10 (23.3)*	*p value*	*CLAD baseline 9 (20.9)*	*CLAD follow-up 9 (20.9)*	*p value*
Myeloid cells % (mean ± SD)												
CD15+/CD33+/CD14-/HLADR−/low (G-MDSC)	58.7 ± 9.4	62.4 ± 12.3	ns	59.9 ± 9.8	67 ± 7.7	**.008**	54.9 ± 10.3	47.2 ± 12.1	ns	59.8 ± 6.7	67.2 ± 9	.06
CD14+/HLADR−/low (Mo-MDSC)	9.8 ± 8.3	5.9 ± 5.6	**.01**	9.6 ± 9.2	5.4 ± 5.2	.06	8.6 ± 5.6	4.6 ± 3.9	.09	11.8 ± 9	8.5 ± 7.7	ns
CD14+CD16+ (Inflammatory monocytes)	11.6 ± 6.5	8.5 ± 4.2	**.01**	11.5 ± 6.2	9 ± 4.5	ns	13.6 ± 7.1	9.2 ± 3.2	.09	9.7 ± 6.9	6.2 ± 3.8	ns
Lymphocytes % (mean ± SD)												
CD3-CD16+ (Natural Killer)	11.9 ± 4.9	14.3 ± 7.7	.08	12.2 ± 4.8	15.1 ± 7.8	ns	12.4 ± 5.4	11 ± 6.1	ns	10.3 ± 4.9	15.6 ± 8.7	ns
T CD3+	74.4 ± 6.8	63.2 ± 10.7	**< .001**	74 ± 6.9	64.2 ± 12.1	**.001**	73.3 ± 7.5	64.2 ± 7.8	**.02**	76.4 ± 6.3	59.3 ± 9.7	**< .001**
CD3+CD4+ (T-helper)	47 ± 6.2	42.2 ± 8.8	**.005**	47.2 ± 5.9	45 ± 8.9	ns	46.9 ± 4.5	43.4 ± 5.1	ns	46.7 ± 8.8	33.6 ± 6.2	**.002**
CD3+CD8+ (T-cytotoxic)	27 ± 7.4	19.5 ± 6.4	**< .001**	26.7 ± 7	18.5 ± 6.4	**< .001**	25.3 ± 7.6	19.7 ± 5.7	.09	29.7 ± 8.4	22.1 ± 7.2	**.05**
CD4+/CD8+ ratio	1.9 ± 0.9	2.4 ± 0.9	**.02**	1.9 ± 0.8	2.6 ± 0.9	**.004**	2.1 ± 0.8	2.4 ± 0.7	ns	1.8 ± 1	1.7 ± 1	ns
CD4+CD45RA+ (T-naïve)	37.2 ± 13.8	44.6 ± 15.4	**.02**	40.3 ± 14.7	51.7 ± 16.8	**.02**	36.1 ± 11.3	35.6 ± 7.8	ns	30.1 ± 11.6	35.5 ± 5.3	ns
CD4+CD161+	15.2 ± 6.9	8.6 ± 4.6	**< .001**	16.2 ± 6.5	7 ± 3.9	**< .001**	12.5 ± 7.9	9.8 ± 4.1	ns	15.4 ± 7.1	11.6 ± 5.3	ns
CD4+CD25+CD127low/− (T-reg)	8 ± 2.4	6.2 ± 2.8	**.002**	8.1 ± 2.5	5.9 ± 3	**.009**	8.1 ± 3.1	6.1 ± 3	ns	7.7 ± 1.6	7 ± 1.9	ns
CD19+ (B cells)	11.2 ± 4.1	15 ± 8.1	**.009**	11.3 ± 4.3	14.3 ± 8.3	ns	9.9 ± 3.1	16.1 ± 8.1	**.04**	12.8 ± 4.2	15.5 ± 8.2	ns
CD19+CD27-IgD+ (B-naïve)	58.7 ± 11.9	66.7 ± 13	**.004**	56.2 ± 11.8	70.5 ± 10.8	**< .001**	62.8 ± 10.7	56.7 ± 11.8	ns	60.8 ± 13.1	67.9 ± 15	ns
CD19+CD27+CD38-IgM-IgD− (switched B-memory)	17.5 ± 7.9	11.9 ± 11.3	**.01**	19.5 ± 9.1	7.8 ± 5.9	**< .001**	15.2 ± 4.1	22.5 ± 16.6	ns	14.3 ± 6.7	11 ± 8.6	ns
CD19+CD27+CD38-IgM+IgD+ (unswitched B-memory)	11.9 ± 5.8	10.1 ± 6.5	ns	11.6 ± 5.5	9.2 ± 5.2	ns	12 ± 7	11.8 ± 7.6	ns	12.8 ± 5.8	10.7 ± 8.6	ns

Abbreviations: CLAD: cladribine; DMF: dimethyl fumarate; EDSS: Expanded Disability Status Scale; IQR: interquartile range; MRI: Magnetic Resonance Imaging; NTZ: natalizumab; SD: standard deviation.

Table S2. Correlations between differences in terms of immunophenotypic cell subsets and clinical/radiological characteristics.

	Follow-up EDSS	Brain follow-up MRI lesions on T2 weighted sequences	Spinal follow-up MRI lesions on T2 weighted sequences	Brain follow-up MRI lesions on T1 gadolinium weighted sequences	Spinal follow-up MRI lesions on T1 gadolinium weighted sequences	Patients with relapses in the months between naïve and follow-up PB sample
Myeloid cells % (mean ± SD)						
CD15+/CD33+/CD14-/HLADR−/low (G-MDSC)	-0.071	-0.042	-0.063	-0.071	-0.069	-0.071
CD14+/HLADR−/low (Mo-MDSC)	-0.101	-0.021	-0.033	-0.023	-0.023	-0.034
CD14+CD16+ (Inflammatory monocytes)	-0.081	-0.041	-0.043	-0.082	-0.067	-0.063
Lymphocytes % (mean ± SD)						
CD3-CD16+ (Natural Killer)	-0.034	-0.002	-0.003	-0.021	-0.021	-0.038
T CD3+	-0.129	-0.006	-0.023	-0.054	-0.041	-0.229
CD3+CD4+ (T-helper)	-0.045	-0.043	-0.063	-0.070	-0.041	-0.059
CD3+CD8+ (T-cytotoxic)	-0.004	-0.009	-0.006	-0.006	-0.004	-0.021
CD4+/CD8+ ratio	-0.075	-0.043	-0.071	-0.077	-0.061	-0.054
CD4+CD45RA+ (T-naïve)	-0.212	-0.065	-0.021	-0.032	-0.046	-0.023
CD4+CD161+	-0.143	-0.065	-0.070	-0.079	-0.043	-0.121
CD4+CD25+CD127low/− (T-reg)	-0.082	-0.072	-0.071	-0.071	-0.069	-0.027
CD19+ (B cells)	-0.201	-0.087	-0.039	-0.056	-0.051	-0.324
CD19+CD27-IgD+ (B-naïve)	-0.093	-0.032	-0.082	-0.039	-0.029	-0.057
CD19+CD27+CD38-IgM-IgD− (switched B-memory)	-0.082	-0.029	-0.033	-0.023	-0.005	-0.063
CD19+CD27+CD38-IgM+IgD+ (unswitched B-memory)	-0.074	-0.056	-0.033	-0.001	-0.071	-0.070

Abbreviations: EDSS: Expanded Disability Status Scale; MRI: Magnetic Resonance Imaging; PB: peripheral blood; SD: standard deviation.

Note: * Pearson coefficient was used for this correlation.
